# *Wolbachia*-Mediated Cytoplasmic Incompatibility Is Associated with Impaired Histone Deposition in the Male Pronucleus

**DOI:** 10.1371/journal.ppat.1000343

**Published:** 2009-03-20

**Authors:** Frédéric Landmann, Guillermo A. Orsi, Benjamin Loppin, William Sullivan

**Affiliations:** 1 Department of Molecular, Cell, and Developmental Biology, University of California Santa Cruz, Santa Cruz, California, United States of America; 2 Centre de Génétique Moléculaire et Cellulaire, CNRS UMR5534, Université Lyon 1, Lyon, France; Stanford University, United States of America

## Abstract

*Wolbachia* is a bacteria endosymbiont that rapidly infects insect populations through a mechanism known as cytoplasmic incompatibility (CI). In CI, crosses between *Wolbachia*-infected males and uninfected females produce severe cell cycle defects in the male pronucleus resulting in early embryonic lethality. In contrast, viable progeny are produced when both parents are infected (the Rescue cross). An important consequence of CI–Rescue is that infected females have a selective advantage over uninfected females facilitating the rapid spread of *Wolbachia* through insect populations. CI disrupts a number of prophase and metaphase events in the male pronucleus, including Cdk1 activation, chromosome condensation, and segregation. Here, we demonstrate that CI disrupts earlier interphase cell cycle events. Specifically, CI delays the H3.3 and H4 deposition that occurs immediately after protamine removal from the male pronucleus. In addition, we find prolonged retention of the replication factor PCNA in the male pronucleus into metaphase, indicating progression into mitosis with incompletely replicated DNA. We propose that these CI-induced interphase defects in *de novo* nucleosome assembly and replication are the cause of the observed mitotic condensation and segregation defects. In addition, these interphase chromosome defects likely activate S-phase checkpoints, accounting for the previously described delays in Cdk1 activation. These results have important implications for the mechanism of Rescue and other *Wolbachia*-induced phenotypes.

## Author Summary

*Wolbachia* are among the most successful of all intracellular bacteria, infecting an estimated 65% of insect species. *Wolbachia* are also present in filarial nematodes and are the cause of African river blindness. *Wolbachia*'s success is due in part to its ability to induce a conditional form of sterility known as cytoplasmic incompatibility (CI), endowing infected females with a tremendous selective advantage. CI results in the severe reduction in progeny from crosses between uninfected females and *Wolbachia*-infected males. However, *Wolbachia*-infected females can mate with either infected or uninfected males with no reduction in progeny. CI may drive speciation and is intensively being pursued as a means to control insect-borne human disease. In spite of its biological and medical significance, the molecular basis of CI is not understood. We take advantage of newly generated chromatin reagents to demonstrate that prior to the well-documented defects in chromosome condensation and segregation, CI produces a delay in recruiting the replication-independent histone H3.3/H4 complex to the male pronucleus. There is great interest in histone H3.3 because of its general role in transcription and in remodeling of the sperm chromatin following fertilization. In addition, these findings may provide insight into other *Wolbachia*–host interactions such as CI–Rescue and male-killing.

## Introduction

*Wolbachia* are intracellular bacteria that infect some 65% of all insect species [1]. Their success is in large part due to their efficient maternal transmission and their ability to alter host reproduction such that infected females produce more offspring than uninfected females [2]. The most common form of altered reproduction is known as cytoplasmic incompatibility (CI), a form of conditional sterility resulting from crosses of *Wolbachia*-infected males to uninfected females [3]. These crosses produce defects in the first zygotic mitosis resulting in inviable embryos. Significantly, if both the female and the male are infected, no defects are observed and viable embryos are produced. This phenomenon is known as Rescue [4]. Consequently in *Wolbachia*-infected populations, infected females produce viable progeny whether they mate to infected or uninfected males. In contrast, uninfected females produce viable progeny only when mated to uninfected males. Thus infected females enjoy a tremendous selective advantage over uninfected females resulting in the rapid spread of *Wolbachia* via the maternal lineage [5]. The success of this strategy is underscored by the fact that CI has been documented in every insect order [3].

CI crosses produce embryos in which the paternal chromosomes are improperly condensed when aligned at the metaphase plate of the first mitotic division following fertilization [6]–[8]. It should be noted that the first mitotic division is unique in many insects, including Drosophila, because the paternal and maternal chromosomes reside on separate regions of the metaphase plate and are independently regulated with respect to entry into anaphase [7],[9]. As the embryo progresses into anaphase, paternal sister chromatids either fail to segregate, or exhibit extensive bridging and fragmentation during segregation, a hallmark of damaged or incompletely replicated chromosomes [9]. It is thought that strong CI elicits chromosome condensation defects severe enough to activate the spindle assembly checkpoint and prevent segregation while weak CI results in more mild defects in which the checkpoint fails to activate, allowing improper segregation [8]. Defects earlier in the cell cycle at the prophase/metaphase transition have also been reported. These include a delay in Cdk1 activation and nuclear envelope breakdown in the male pronucleus relative to the female pronucleus [10].

These observations leave unresolved the cause and effect relationship between the chromosome condensation and Cdk1 activation defects in CI embryos. It is well established that defects in DNA replication and chromosome condensation lead to cell cycle checkpoint induced delays in Cdk1 activation [11]. However Cdk1 activation is required to drive chromosome condensation and failed Cdk1 activation results in failed chromosome condensation [12]. To identify the proximal defects in CI embryos, we sought to determine whether CI-induced chromatin defects occur prior to Cdk1 activation during the interphase/prophase transition. Identification of earlier chromatin defects, during the sperm to male pronucleus transformation, would strongly argue that these are proximal to and the cause of the delayed Cdk1 activation and chromosome condensation/segregation defects observed during prophase and metaphase.

Based on this reasoning, the work presented here focuses on sperm formation and sperm transformation into the male pronucleus in normal and CI crosses. To facilitate a compact configuration, the sperm chromatin is packaged with specialized small basic proteins known as protamines [13]. Another unique property of the *Drosophila* sperm is that the nuclear envelope lacks lamins and nuclear pores [14]. Immediately following fertilization, the nuclear envelope, the plasma membrane and the protamines are removed, and *de novo* nucleosome assembly is initiated using maternally supplied core histones [15]. This nucleosome assembly occurs prior to DNA replication, and is executed by a replication-independent pathway that uses histone variant H3.3 and its specific chaperone HIRA [15]. In addition, the formation of the male pronucleus requires the ATP-dependent chromatin remodeling enzyme CHD1 [16]. After these remodeling events, the nucleus acquires a conventional nuclear envelope containing lamins and nuclear pores. As the egg completes meiosis, the newly formed male and female pronuclei initiate DNA replication while migrating towards one another. Once the replication is complete, Cdk1 activation triggers mitotic entry in the closely apposed pronuclei [17].

The studies presented here demonstrate CI- specific defects in H3.3/H4 deposition and prolonged retention of PCNA in the male pronucleus. These results suggests that in CI crosses, the male pronucleus enters mitosis with improperly condensed chromatin and incompletely replicated DNA. Significantly remodeling of the sperm chromatin including protamine removal and H3.3/H4 deposition occurs during interphase, well before Cdk1 activation and entry into mitosis. Thus our results suggest a model in which the initial defects in chromatin assembly in the male pronucleus activate cell cycle checkpoints delaying Cdk1 activation and mitotic entry. These chromatin remodeling defects also explain previous findings of defects during metaphase and anaphase in chromatin condensation and segregation. Because H3.3 deposition plays a key role in the transcriptional regulation throughout development, our results may provide insight into other effects *Wolbachia* has on its host.

## Results

### CI–Induced Defects Are Limited to Paternal Chromosomes

**Figure 1:**
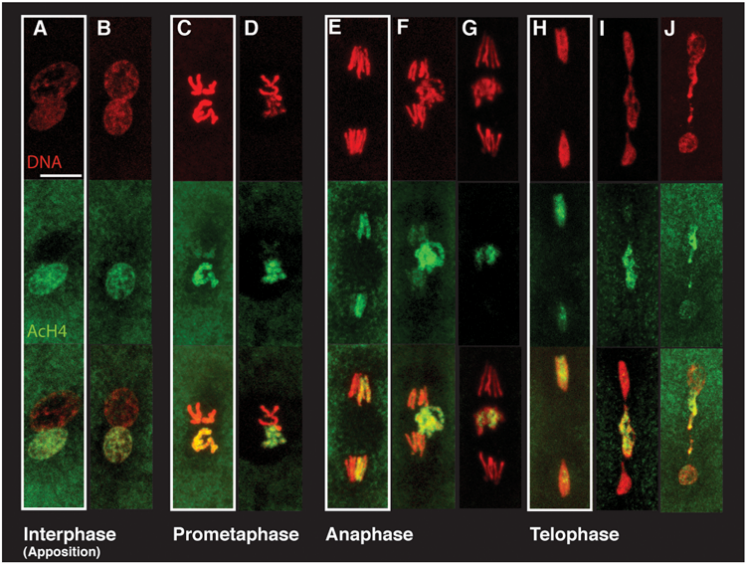
In *D. simulans* embryos from incompatible crosses (CI), paternal chromosomes fail to condense and improperly segregate during the first mitosis. (A,C,E,H) are uninfected controls in white boxes. (B,D,F,G,I,J) are CI embryos. Paternal, but not maternal chromosomes incorporate acetylated histone H4 during *de novo* nucleosome assembly (green). DNA is detected with propidium iodide (red). (A,B) pronuclear apposition. (C,D) prometaphase. (E,F,G) anaphase A (F) or B (E,G). (H,I) telophase. (J) late telophase/second S phase. Scale bar is 5 µm.

To confirm that the CI-induced segregation and condensation defects are limited to the paternal chromosomes, we used an antibody directed against acetylated histone H4 that preferentially labels the *de novo* assembled paternal chromatin after protamine removal in *Drosophila* eggs (Figure 1, [15]). We used *D. simulans* rather than *D. melanogaster*, since CI is very robust in the former species only. In CI embryos, the maternal chromosomes segregate normally at anaphase while the paternal chromosomes lag on the metaphase plate. At late telophase, bridges are observed between separating paternal sister chromosome complements (Figure 1, [7]). This results in severe nuclear division failures and accounts for the pre-cellular embryonic lethality in CI crosses. In stronger CI cases, severe disruption of paternal chromosome segregation results in their exclusion from both daughter nuclei. In haplo-diplo species this pattern of segregation produces viable haploid males [8]. The detection of acetylated histone H4 also demonstrates that sperm chromatin remodeling is initiated in CI crosses and this led us to examine protamine removal and histone deposition during this period.

### Protamine Removal Appears Normal in CI Embryos

**Figure 2:**
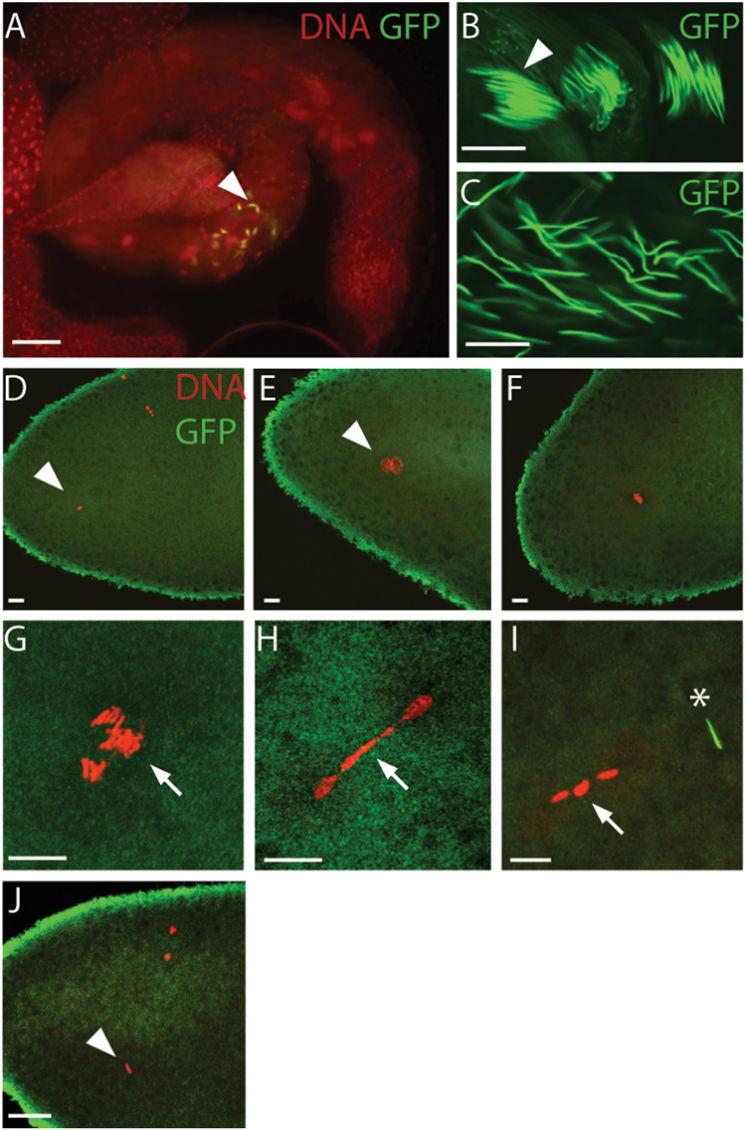
Protamine incorporation and removal appear normal in *D. simulans* CI crosses. (A,B,C) In infected *D.simulans* transgenic male testis, Protamine-GFP is detected in groups of late spermatid nuclei (arrowheads in A and B) and in sperm nuclei in seminal vesicles (C). (D,E,F,G,H) Confocal sections of embryos from non-infected females crossed with infected, transgenic males. Protamine-GFP is never detected in the male nucleus (arrowhead) as early as the second female meiotic division (D) or at the pronuclear apposition stage (E). (F,G,H,I) Cycle 1 embryos in metaphase (F), anaphase (G) or telophase (H,I). The embryos in G–I display an obvious CI phenotype with lagging paternal chromatids or chromatin bridges (arrows). No Protamine-GFP is detected in the late paternal chromatin. (I) embryo containing a second, non-activated sperm nucleus (asterisk) whose Protamine-GFP has not been removed serving as internal control for Protamine-GFP detection in embryos. (J) Embryo from non-infected females crossed with non-infected transgenic males. Protamine-GFP is never detected in the male nucleus (arrowhead) in this control. DNA is stained with propidium iodide (red) in all panels except B and C. GFP is detected either directly (A,B,C) or with the use of an anti-GFP antibody (green) (D,E,F,G,H,I,J). Scale bar is 50 µm in A and 10 µm in all other panels.

During spermatogenesis in many higher eukaryotes, including *Drosophila*, core histones in the sperm nuclei are replaced by protamines, sperm-specific chromosomal proteins that allow a greater chromatin compaction [18]. To assay protamine deposition and removal in CI embryos, we created a transgenic *D. simulans* stock expressing *D. simulans* protamine fused to GFP under the control of its endogenous promoter. In non-infected and infected testis, the fusion protein was incorporated into spermatids and present in mature sperm in seminal vesicles. (Figure 2A, 2B, and 2C). In both, control and CI fertilized embryos, Protamine-GFP was removed immediately after sperm entry, before completion of the female meiotic division (Figure 2, n = 22 for CI (D–H), n>20 for control (J)). To verify that Protamine-GFP can be visualized in early *D. simulans* embryos, we took advantage of rare double fertilization events (Figure 2I, asterisk). In this case Protamine-GFP was visible in the additional, non-activated sperm DNA while absent from the male chromosomes lagging on the metaphase plate (arrow). Thus, at the cytological level, no obvious differences in protamine removal and deposition are observed in CI embryos.

### CI Affects Histone Deposition in the Male Pronucleus

Immediately following the removal of protamines from the male pronucleus, paternal nucleosomes are assembled using maternally supplied histones. This replication-independent nucleosome assembly specifically involves the H3.3 histone variant, which is deposited along with H4, followed by H2A and H2B [19]. H3.3 is thus specifically deposited in the male pronucleus before the completion of the female meiosis and remains enriched in paternal chromosomes throughout the first mitotic division. The paternal chromosomes lose H3.3 by incorporation of canonical histone H3 with each new round of replication [20].

**Figure 3:**
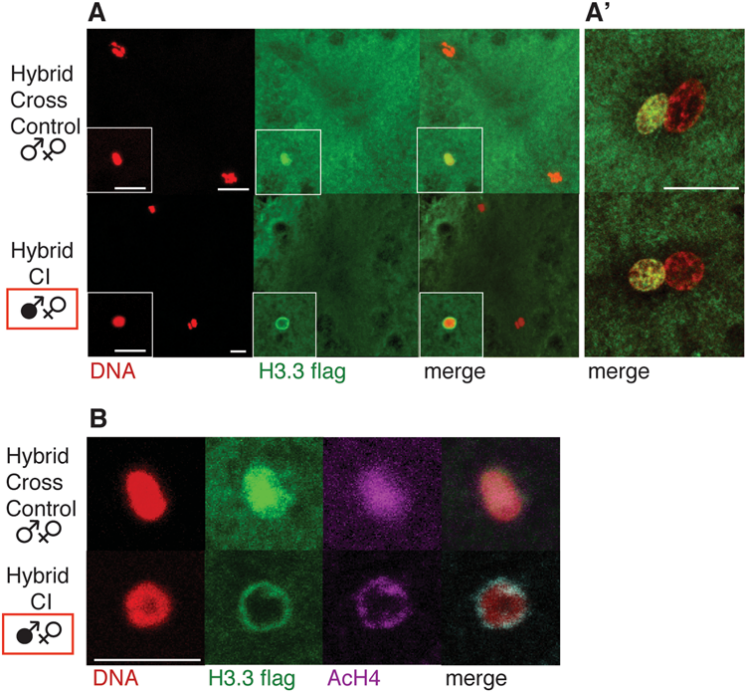
Histone variant H3.3 deposition is abnormal in CI *D. melanogaster* / *D. simulans* hybrid crosses. (A) Embryos from hybrid control (uninfected *D. melanogaster* females x uninfected *D. simulans* males) or CI (uninfected *D. melanogaster* females x infected *D. simulans* males) crosses were stained to reveal a tagged H3.3 (green) and DNA (propidium iodide in red), after sperm entry. The two female meiotic products are still in metaphase II, indicating that sperm entry just occurred (in white frame). (A′) H3.3 deposition is undistinguishable between embryos from hybrid control or CI crosses during pronuclear apposition. Note that the male pronucleus is always slightly smaller then the female pronucleus. (B) Acetylated histone H4 colocalizes with H3.3 in perinuclear rings in CI. Magnification of male pronuclei from hybrid crosses, acetylated H4 in purple. Scale bar is 10 µm.

In order to take advantage of both the strong CI of *D. simulans* and of transgenic markers only available in *D. melanogaster*, we performed hybrid crosses between *D. simulans* males and *D. melanogaster* females. Previous studies demonstrated that this hybrid cross exhibits a robust CI and Rescue and is an appropriate system for studying CI [21]. Infected or non-infected *D. simulans* males were crossed with non-infected transgenic *D. melanogaster* females expressing a tagged H3.3-FLAG histone (CI and control crosses, respectively). In all embryos examined from the above control hybrid cross (n = 51), a robust H3.3 deposition was observed in the male pronucleus prior to completion of female meiosis, similar to the H3.3 deposition observed in single species *D. melanogaster* control crosses (not shown). All exhibited normal H3.3 deposition in the male pronucleus before the completion of female meiosis (n = 30, Figure 3A). However in hybrid CI crosses, 22% of the embryos exhibited an abnormal H3.3 accumulation at the periphery of the male pronucleus before the completion of female meiosis (n = 63, Figure 3A). In all nuclei with an abnormal accumulation at the periphery, no H3.3 staining was observed inside the nucleus suggesting a failure or an altered pattern of early H3.3 deposition. No lamin is detected at this stage (Figure S1), which suggests that nucleosome assembly occurs prior to the formation of the pronuclear envelope, ruling out a general nuclear import defect. Double immunostaining experiments showed that histone H4 colocalized with H3.3 in peripheral rings in CI embryos (Figure 3B). These abnormal rings of H3.3 and H4 are never observed during pronuclei apposition (Figure 3A′, n>30 for control and CI crosses). This suggests that CI results in a delayed, but not complete inhibition of H3.3/H4 nuclear deposition.

### CI Affects Male Pronuclear DNA Replication

**Figure 4:**
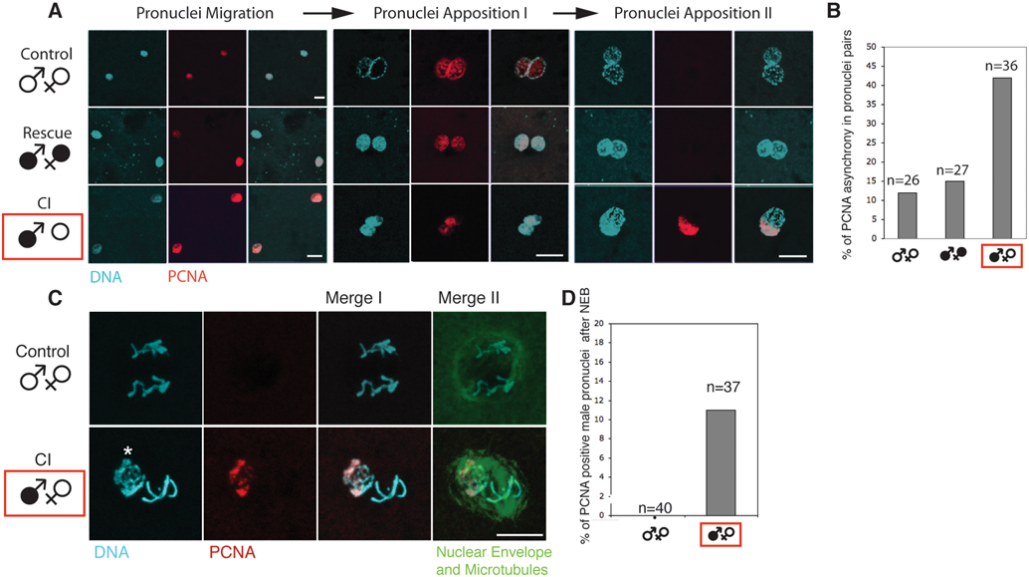
In *D. simulans*, replication of the male pronucleus is prolonged in CI embryo. (A) Embryos from control, rescue, or CI crosses were fixed and stained for PCNA (red), and DNA (propidium iodide, cyan). Scale bars are 10 µm. (B) Synchrony was scored when both apposed pronuclei were PCNA negative. Conversely, asynchrony was established when a pronucleus was PCNA positive whereas its counterpart was negative. (C) In CI embryo, PCNA is present in male pronuclear chromatin after pronuclear envelopes breakdown and spindle assembly. Embryos from control and CI crosses were fixed and stained for PCNA, and with two monoclonal antibodies, the anti-lamin ADL84 and an anti-tubulin to reveal the presence of the pronuclear envelopes and the spindle set up respectively (in green). The asterisk marks the uncondensed male pronucleus. Scale bar is 10 µm. Male pronuclei can be identified according to their smaller size compare to female pronuclei during apposition (A), or because of the chromosome condensation defects in CI (C). (D) % of PCNA positive male pronuclei after NEB in control crosses and CI crosses.

Once the paternal chromatin is assembled with maternally supplied core histones including H3.3 and H4, the DNA must replicate prior to mitotic entry in both pronuclei. We examined replication timing of pronuclei in control and CI embryos using an antibody directed against the *Drosophila* Proliferating Cell Nuclear Antigen (PCNA). PCNA is a conserved core component of the replication fork [22] and only present in S-phase nuclei [23]. To confirm this specificity in *Drosophila*, we examined PCNA localization in early embryos where the S-phase is well characterized with respect to chromosome and spindle morphology [24] (Figure S2). These studies demonstrate that PCNA is nuclear only during S-phase, confirming previous results. Early *D. simulans* embryos from uninfected and CI crosses were examined from the time of pronuclear migration to pronuclear apposition. In the uninfected crosses, both the male and female pronuclei exhibit robust PCNA staining during their migration, indicating that the S-phase is initiated during the early stages of pronuclei migration (Figure 4A, n>30). We always observed synchronous PCNA staining in both nuclei, indicating simultaneous S-phase initiation in the male and female pronuclei. During pronuclei apposition in the uninfected crosses, we either observe that both pronuclei possess (Figure 4A, “apposition I”) or lack PCNA staining (Figure 4A, “apposition II”). S phase was completed during pronuclear apposition and not earlier. S phase was completed synchronously between male and female pronuclei in 88% of embryos (n = 26, Figure 3A and 3B). We performed the same analysis in embryos derived from the Rescue cross. The results for both pronuclear migration and apposition were very similar to the control cross (n = 27, Figure 4A and 4B).

Next, we analyzed PCNA staining in embryos derived from the CI cross. As with the control cross, both pronuclei stained positive for PCNA throughout migration (Figure 4A, n>30). Thus, like the control cross, S-phase is initiated simultaneously in the male and female pronuclei during the initial stages of pronuclear migration. Unlike the control crosses, however, we observed 43% of embryos (n = 36) with differential staining during apposition (Figure 4A and 4B). These results indicate that CI delays completion of replication in the male pronucleus. Because the timing of replication initiation does not appear to be altered in CI embryos, it is likely that the replication is slowed down or blocked in the male pronucleus of CI embryos relative to control embryos. Alternate interpretations include delayed release of PCNA or extra DNA replication in CI embryos. However delayed Cdk1 activation in the male pronucleus, presumably due to activation of cell cycle checkpoints, favors a model in which of disrupted replication in the male pronucleus of CI embryos.

### CI Embryos Enter the First Zygotic Mitosis with Replication-Associated Defects in the Paternal Chromosomes

We also examined PCNA staining in control and CI *D. simulans* embryos that had progressed into prophase as evidenced by condensed DNA, spindle formation, and NEB. In control embryos, PCNA was never localized in the pronuclear DNA after NEB (n = 40, Figure 4C). In CI embryos however, 11% of pronuclei pairs observed after NEB showed a PCNA staining associated with the poorly and unevenly condensed male pronuclear DNA (n = 37, Figure 4C and 4D). Once the male pronuclei of CI embryos progress into metaphase, we no longer observe such PCNA staining.

It has been reported that PCNA is associated with damaged as well as replicating DNA (for a review see [25]). We favor a replication defect to explain CI rather than DNA breaks, given that chromatin remodeling defects are strongly associated with replication defects [26]. In addition, chromosome bridging during the first telophase but not free chromosome fragments is well documented in CI embryos. This is more consistent with DNA replication rather than damage defects. Taken together, our data suggest that in CI embryos DNA replication is slowed down or blocked in the male pronucleus.

## Discussion

Genetic and cellular analyses indicate that CI specifically disrupts paternal chromosome condensation, congression and segregation [9],[27]. Here we take advantage of anti-acetylated H4 histone antibodies that specifically stain the paternal chromosomes due to nucleosome assembly in the male pronucleus. This enabled us to directly demonstrate the effects of CI are limited to the paternal chromosomes. This implies that CI targets processes specific to the paternal chromosomes necessary for progression through mitosis.

To identify these processes, we focused on the chromosome remodeling events that are specific to sperm formation and transform the sperm into a male pronucleus. Our cytological examination of protamine deposition and removal did not reveal obvious abnormalities in CI embryos. This of course does not rule out more subtle defects. Protamines are normally removed immediately following fertilization and replaced with the replication-independent variant histone H3.3 and canonical H4, H2A/H2B histones. In CI embryos, a significant fraction of embryos exhibit delays in H3.3 incorporation before completion of the female meiosis. This results in an abnormal ring of H3.3 encompassing the male pronucleus. There is no nuclear envelope present at this early stage, indicating the H3.3 ring phenotype is not due to defects in nuclear import. More likely it is due to a delay in loading H3.3 onto the paternal chromosomes.

These CI-induced defects in H3.3 deposition are strikingly similar to those reported for mutants in the chromatin remodeling protein CHD1. Male pronuclei from *chd1* mutants also exhibit an improper accumulation of H3.3 around the male pronucleus. Like the CI-induced defects, chromosome condensation is severely disrupted presumably due to defects in H3.3-based chromatin remodeling [16]. Mutations affecting HIRA, the H3.3 chaperone, also prevent the formation of condensed paternal chromosomes [15]. These replication-independent histone deposition defects can explain the chromosome condensation and segregation defects observed in CI embryos since H3.3 and H3 share a conserved N terminal tail, whose phosphorylation is crucial for chromosome condensation [28]. Defects in histone deposition can also explain the delayed progression through S phase, as proper nucleosome assembly is required for DNA replication [29]. Both replication dependent and independent nucleosome assembly machineries share common interactors, like the histone chaperone ASF1 [19]. ASF1 siRNA knock down experiments and mutants clearly show DNA replication defects [26]. Late DNA replication in ORC2 (Origin Recognition Complex 2) mutants also provoke chromosome condensation defects and reveals that proper replication timing is crucial for the chromatin to be fully competent to condense [30]. However it should be pointed out that chromosome condensation defects alone can produce segregation defects [31].

In addition to playing a role in paternal chromatin remodeling, H3.3 plays a more general role in transcription regulation. The replication-independent deposition of H3.3 is correlated with active chromatin states [32]. This raises the intriguing possibility that *Wolbachia* may influence the transcription state of its host nuclei by altering H3.3 deposition. It has been shown that *Wolbachia* do not influence the *in vivo* expression level of antimicrobial peptides specifically [33], but microarray data from *Drosophila* cell culture suggest that *Wolbachia* has some influence on host transcript levels [34]. Another alteration of the host reproduction caused by *Wolbachia* is a phenomenon called male killing (MK) [35]. In male killing, *Wolbachia* infection results in death of the male but not the female progeny. The resulting increase in the proportion of female progeny is beneficial to the maternally transmitted *Wolbachia*. Moving a specific *Wolbachia* strain from one *Drosophila* species to another results in an instantaneous transition from CI to MK, indicating that these *Wolbachia*-induced phenotypes share a common molecular mechanism [36]. Studies in *Drosophila* demonstrate that disruptions in some chromatin remodelers have a much greater impact on organization of the X chromosomes in males than females [37]. This raises the possibility that CI and MK evolved from *Wolbachia* having a more general effect on the transcriptional state of its host cell by regulating H3.3 deposition.

**Figure 5:**
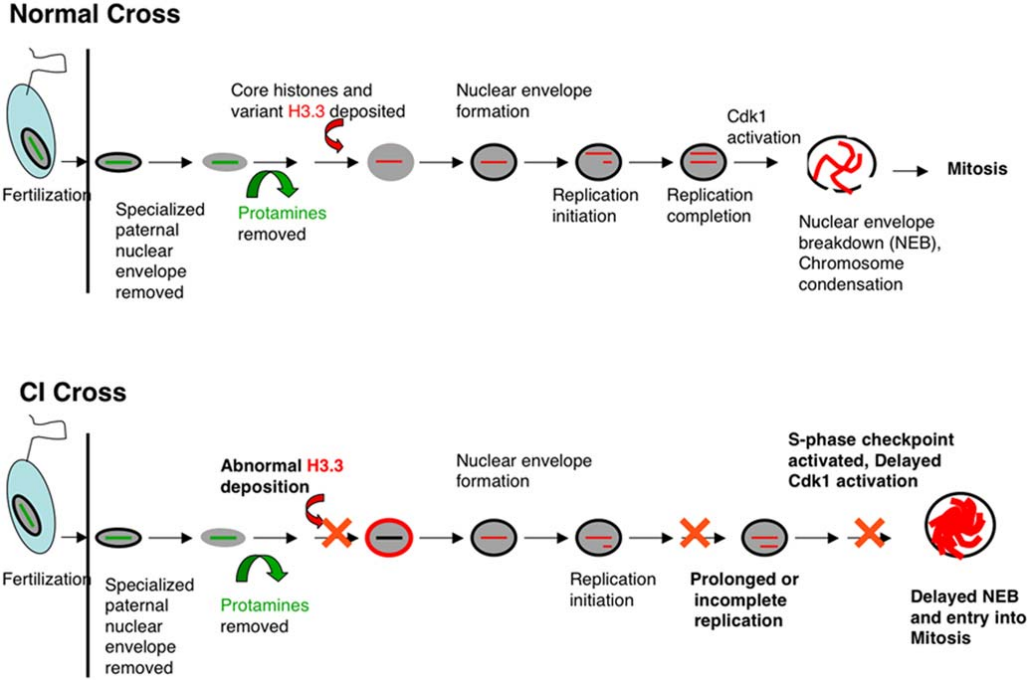
A schematic of key events in the transformation of sperm to male pronucleus in embryos from normal and CI crosses. Normal cross: Immediately following fertilization, the specialized nuclear envelope (lacking nuclear pores) of the male pronucleus is removed. Next, the protamines are removed and replaced by maternally supplied histones, including the replication-independent histone H3.3. This event is followed by lamin deposition and formation of a conventional nuclear envelope containing nuclear pores. Next, S-phase is initiated and upon completion, Cdk1 is activated driving nuclear envelope breakdown, chromosome condensation, and spindle assembly. CI cross: At the cytological level, removal of the sperm nuclear envelope and protamines appear normal. Often however, histone H3.3 deposition is abnormal, resulting in a ring of histone H3.3 encompassing the paternal pronucleus. This is the earliest documented CI phenotype in embryos and is similar to that observed for mutants in the chromatin remodeling protein Chd1. Imaging PCNA, a marker for replicating chromosomes, indicates that replication initiates normally in CI embryos, but is prolonged or incomplete. This may be a direct result of the earlier defects in H3.3 deposition. Replication delays activate S-phase checkpoints and thus are likely the cause of the previously described delays in Cdk1 activation and nuclear envelope breakdown.

To determine whether CI influences replication we monitored for the presence of PCNA, an indicator of replicating DNA, in the male and female pronuclei. This analysis demonstrates that in normal embryos, both initiation and completion of DNA replication occur simultaneously in the two pronuclei. In CI embryos while we find replication is initiated simultaneously, completion of replication is significantly delayed in the male pronucleus. In fact we observe instances of PCNA positive paternal chromosomes during metaphase of the first zygotic division. It is likely that the chromatin remodeling defects described above are responsible for the replication delays of the male pronucleus (see Figure 5). These delays readily account for the extensive chromosome bridging observed during anaphase: segregation of unreplicated chromosomes creates bridges [38],[39].

Delayed completion of replication of the paternal chromosomes provided an opportunity to more precisely determine the timing of CI rescue. Previous studies demonstrated that in the Rescue cross, the chromosome condensation defects at metaphase and segregation defects at anaphase are no longer observed [27]. Additional studies demonstrated that in CI crosses, activation of Cdk1, a highly conserved kinase that drives cells into mitosis [40] in the male pronucleus, is delayed relative to its activation in the female pronucleus [10]. These studies also demonstrated that in Rescue crosses, Cdk1 activation in the male and female pronuclei is synchronous. These studies raise the possibility that Rescue is achieved through correction of cell cycle defects in the male pronucleus. Alternatively, synchrony may be restored by a compensatory slowing of the female pronucleus cell cycle. Our data demonstrate that in Rescue crosses, we no longer observe a discordance in the state of PCNA staining in the male and female pronuclei, indicating the events mediating Rescue occur during interphase prior to Cdk1 activation during prophase. However, these studies do not resolve whether it is due to normalization of the interphase events in the male pronucleus or compensating delay in the female pronucleus. Evidence for the former alternative comes from our observation that unlike CI crosses, in Rescue crosses we never observe PCNA positive chromosomes after entry into metaphase in CI embryos.

## Materials and Methods

### Immunofluorescence and Microscopy

Embryos were collected every 15 minutes and immersed in a pure bleach solution for few seconds to remove the chorion. Next they were washed in distilled water and fixed by vigorous shaking in a 1∶1 heptane/methanol mix. RNAse A (Sigma) treatment was performed for 3 hours at 37°C (10 mg/mL). Primary and secondary antibodies were diluted in PBS+ 0.2% Tween+ 2% BSA. Embryos were incubated overnight at 4°C with primary antibodies. For secondary antibodies, the embryos were incubated at 37°C for three hours.

The following antibodies were used: Polyclonal anti-*Drosophila* PCNA (1∶300), polyclonal (1∶1000) and monoclonal (ADL84, 1∶50) anti- *Drosophila* Lamin (all kindly provided by Paul Fisher), monoclonal anti-alpha tubulin (1∶500, Molecular Probes), polyclonal anti-GFP (1∶500, Chemicon), monoclonal anti-FLAG M2 antibody from Sigma was used to detect flagged H3.3 at 1∶2000, polyclonal anti-acetylated H4 (1∶300, Upstate). Cy5 goat anti-rabbit IgG and Alexa Fluor 488 goat anti–mouse IgG antibodies were used at 1∶150 (Invitrogen). DNA was detected with propidium iodide (Molecular Probes, 1.0 mg/mL solution) after a 20 minute incubation in PBS (1∶50) and a 5 minute wash. To better observe pronuclei deep within the cytoplasm, embryos were cleared and mounted in a (2∶1) benzyl benzoate and benzyl alcohol solution.

Confocal microscope images were captured on an inverted photoscope (DMIRB; Leitz) equipped with a laser confocal imaging system (TCS SP2; Leica) using an HCX PL APO 1.4 NA 63 oil objective (Leica) at room temperature.

### Fly Stocks

*D. simulans* stocks were used as *Wolbachia riverside*-infected or cured. *D. melanogaster* stocks were used as cured. The *Wolbachia* infection status of the stocks was established by both PCR [41] and Propidium iodide staining of fixed reproductive tissues.

### Transgenic Lines

We used the previously described PW8-His3.3-Flag [15]. To construct the PW8-ProtSim-GFP transgene, a *D. simulans* protamine gene was amplified from genomic DNA using the following pair of primers:

Primer Protamine simulans 1: GGGAATTCATGCAAATGCCACACCTCCTCAGTC
Primer Protamine simulans 2: TTGGATCCTTGTTGCAACAAACCCGTCGGCGCT

This PCR fragment was cloned in the PW8 vector in frame with EGFP at the 3′ end of the protamine coding sequence. A homozygous viable and fertile transgenic *PW8-ProtSim-GFP* stock was obtained by P-mediated germline transformation of a *D. simulans white* stock (a gift from Elgion Loreto).

## Supporting Information

Figure S1 Histone H3.3 deposition occurs prior to nuclear envelope formation. Male pronuclei from compatible or CI crosses were scored for the presence of lamin to time Histone H3.3 deposition with respect to nuclear envelop formation. In control crosses we observe H3.3 deposition prior to the association of lamins with the nuclear envelope indicating H3.3 deposition occurs prior to nuclear envelop formation. The same experiment performed in CI crosses reveals that in every instance that we observe an abnormal ring of H3.3 staining the lamins are not present. This suggests that a nuclear envelope has not been formed and that the CI induced defect in H3.3 deposition are not likely due to defects in nuclear import. The lamin becomes clearly visible when the male and female pronuclei are migrating towards each other (data not shown). In CI crosses, one third of the male pronuclei showed a peripheral H3.3 accumulation, and none of them showed cortical lamin (n = 16). Scale bar is 1 µm.
(1.20 MB TIF)

Figure S2 PCNA is only detected in interphase nuclei at cycle 10. Embryos at cycle 10 were stained with the anti drosophila PCNA (red), anti-lamin and anti-tubulin (green) were used to follow the nuclear envelope and the microtubule spindle respectively. DNA (blue) was revealed with propidium iodide. (S) S phase, (Pro) prophase, (Meta) metaphase, (Ana) anaphase, (Telo) telophase.
(2.34 MB TIF)
