# Supplementary figures and images for: Wolbachia-Mediated Cytoplasmic Incompatibility Is Associated with Impaired Histone Deposition in the Male Pronucleus
Source: PLoS Pathog. 2009 Mar 20;5(3):e1000343. doi: 10.1371/journal.ppat.1000343 (PMC2652114; doi:10.1371/journal.ppat.1000343)

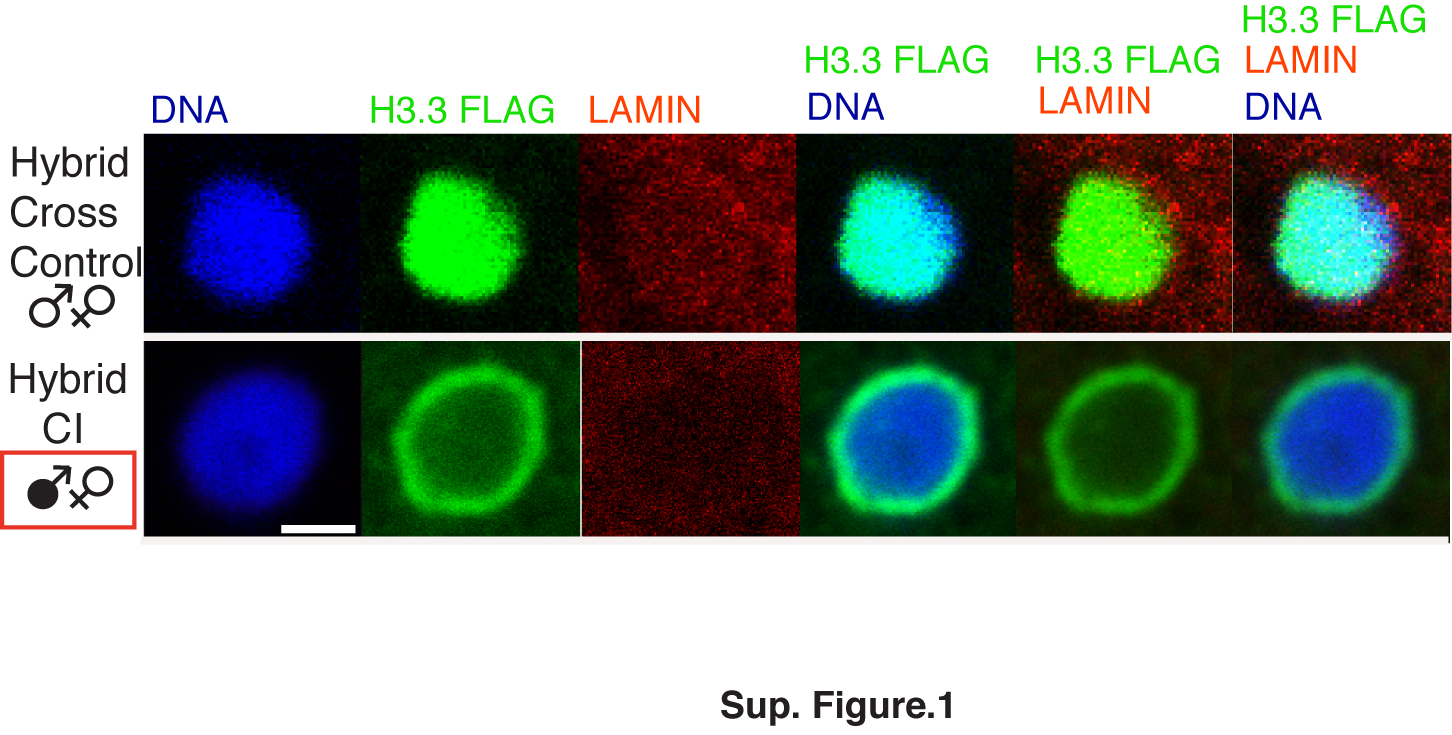

Supplement: Figure S1 — Histone H3.3 deposition occurs prior to nuclear envelope formation. Male pronuclei from compatible or CI crosses were scored for the presence of lamin to time Histone H3.3 deposition with respect to nuclear envelop formation. In control crosses we observe H3.3 deposition prior to the association of lamins with the nuclear envelope indicating H3.3 deposition occurs prior to nuclear envelop formation. The same experiment performed in CI crosses reveals that in every instance that we observe an abnormal ring of H3.3 staining the lamins are not present. This suggests that a nuclear envelope has not been formed and that the CI induced defect in H3.3 deposition are not likely due to defects in nuclear import. The lamin becomes clearly visible when the male and female pronuclei are migrating towards each other (data not shown). In CI crosses, one third of the male pronuclei showed a peripheral H3.3 accumulation, and none of them showed cortical lamin (n = 16). Scale bar is 1 µm. (1.20 MB TIF) [file ppat.1000343.s001.tif]

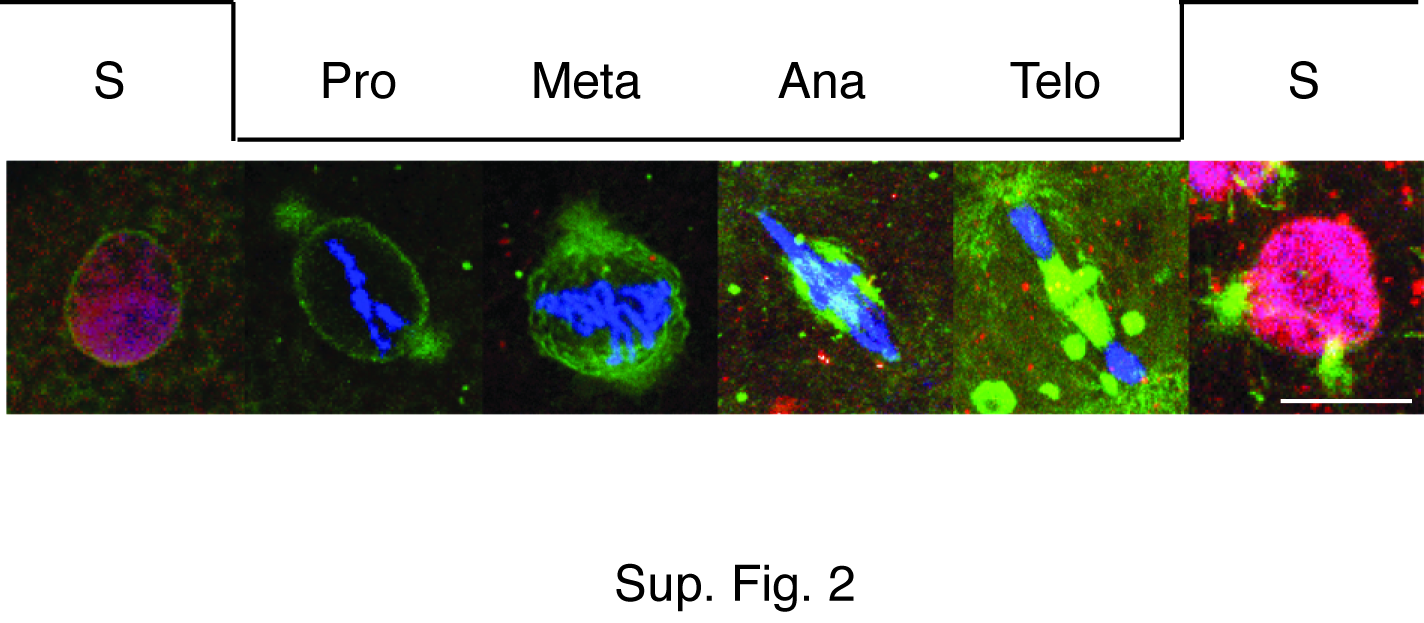

Supplement: Figure S2 — PCNA is only detected in interphase nuclei at cycle 10. Embryos at cycle 10 were stained with the anti drosophila PCNA (red), anti-lamin and anti-tubulin (green) were used to follow the nuclear envelope and the microtubule spindle respectively. DNA (blue) was revealed with propidium iodide. (S) S phase, (Pro) prophase, (Meta) metaphase, (Ana) anaphase, (Telo) telophase. (2.34 MB TIF) [file ppat.1000343.s002.tif]
